# Virtual reality facial emotion recognition in social environments: An eye-tracking study

**DOI:** 10.1016/j.invent.2021.100432

**Published:** 2021-07-17

**Authors:** C.N.W. Geraets, S. Klein Tuente, B.P. Lestestuiver, M. van Beilen, S.A. Nijman, J.B.C. Marsman, W. Veling

**Affiliations:** a Department of Psychiatry, University of Groningen, University Medical Center Groningen, Groningen, the Netherlands; b Department of Psychotic Disorders, GGZ-Drenthe, Assen, the Netherlands; c Cognitive Neuroscience Center, Department of Biomedical Sciences of Cells, University of Groningen, University Medical Center Groningen, Groningen, the Netherlands

**Keywords:** Virtual reality, Emotion recognition, Eye-tracking, Affect, Emotion, Avatars

## Abstract

**Background:**

Virtual reality (VR) enables the administration of realistic and dynamic stimuli within a social context for the assessment and training of emotion recognition. We tested a novel VR emotion recognition task by comparing emotion recognition across a VR, video and photo task, investigating covariates of recognition and exploring visual attention in VR.

**Methods:**

Healthy individuals (n = 100) completed three emotion recognition tasks; a photo, video and VR task. During the VR task, emotions of virtual characters (avatars) in a VR street environment were rated, and eye-tracking was recorded in VR.

**Results:**

Recognition accuracy in VR (overall 75%) was comparable to the photo and video task. However, there were some differences; disgust and happiness had lower accuracy rates in VR, and better accuracy was achieved for surprise and anger in VR compared to the video task. Participants spent more time identifying disgust, fear and sadness than surprise and happiness. In general, attention was directed longer to the eye and nose areas than the mouth.

**Discussion:**

Immersive VR tasks can be used for training and assessment of emotion recognition. VR enables easily controllable avatars within environments relevant for daily life. Validated emotional expressions and tasks will be of relevance for clinical applications.

## Highlights

• Emotion recognition was comparable in the virtual reality (VR), film and photo task.
• Eye-tracking showed emotion-related differences in attention to the face in VR.
• Findings support the utility of VR emotional stimuli for assessment and training.

## 1 Introduction

Identification of facial emotional expressions is crucial for everyday social functioning. Impairments in facial affect recognition have been found among patients with neurological and psychiatric disorders (Henley et al., 2012; Griffiths et al., 2019; Kohler et al., 2011; Savla et al., 2013; Dalili et al., 2015). Therefore, emotion recognition tasks are key for assessment and training to improve social cognition and functioning (Horan and Green, 2019). Recently, immersive virtual reality (VR) has emerged as a promising method.

Conventional emotion recognition tasks using photographs or videos have several disadvantages: stimuli cannot be manipulated easily to adapt task difficulty (Calvo and Nummenmaa, 2016). Furthermore, most stimuli show the isolated face or upper body only on white or neutral backgrounds. Moreover, people are not *present* within the situation because they are looking at 2D computer screens or photographs. In contrast, emotion recognition in daily life takes place within complex environments with distractions, and often during interactions. Therefore, conventional tasks are limited in capturing the complexity of emotion recognition in real life.

VR-based assessment and training materials may offer a solution; virtual faces are dynamic, adaptable and enable interactive practice (Grabowski et al., 2019; Nijman et al., 2019; Nijman et al., 2020). Research using implicit measures has shown that immersive VR can be used to elicit emotions (Marín-Morales et al., 2020). In VR, emotional stimuli can be presented in relevant 3D environments, resembling situations in which emotion recognition takes place in daily life. For instance, the physical surroundings, noise, crowdedness of the environment, and the appraisal of a situation can influence emotion recognition by distracting and capturing attention. In people with cognitive impairments or attentional deficits, in particular, the environment may influence emotion recognition (Wieser and Brosch, 2012). In psychotic or anxiety disorders, environmental factors may affect attention and perception due to greater sensitivity to sensory stimuli, hypervigilance, reduced information processing speed, or situation-induced fear (Wieser and Brosch, 2012; Mühlberger et al., 2008; Nikolaides et al., 2016; Sasson et al., 2007; Sasson et al., 2016).

Deviations in visual attention for faces and social scenes have been observed in various disorders, such as psychosis, social anxiety, conduct disorders and autism spectrum disorders (Griffiths et al., 2019; Dechant et al., 2017; Toh et al., 2011; Martin-Key et al., 2018). Eye-tracking research in psychosis showed restricted scanning of faces, characterized among other things by avoidance of salient facial features (eyes, nose and mouth) (Toh et al., 2011). People with autism were found to direct less attention to faces when more people are present, which contrasts with typically developing adults (Guillon et al., 2014). Furthermore, socially anxious people were found to focus more on avatars' bodies and the environment than faces, while performing social interaction tasks in a VR train (Dechant et al., 2017).

The validity of isolated static and dynamic 2D virtual faces has been shown previously in healthy populations and people with a psychotic disorder (Gutiérrez-Maldonado et al., 2013; Dyck et al., 2008; Dyck et al., 2010; Gutiérrez-Maldonado et al., 2012). These studies reported emotion recognition accuracy to be similar for real and virtual faces, with happiness being recognized best. Negative emotions, such as sadness, anger and disgust were the most difficult to identify. However, much remains unknown about emotion perception in immersive 3D VR.

We investigated a novel immersive VR emotion recognition task intended for assessment and training. This was done by 1] comparing recognition accuracy with two conventional tasks (Young et al., 2002; Bryson et al., 1997), 2] exploring covariates (age, sex, education and VR environmental distractors in terms of street crowding), and 3] determining visual attention with eye-tracking in VR.

We expected small advantages for females and those with a higher education level (Bediou et al., 2007; Kret and De Gelder, 2012; Meletti et al., 2009; Kessels et al., 2014), and stronger age-related declines for virtual than real faces due to more computer exposure in younger people (Dyck et al., 2008). We expected that environmental distractors would lower the accuracy and speed of emotion recognition. Concerning visual attention, it was hypothesized that in virtual faces, like real faces, most attention is focused on the eyes (Wells et al., 2016), and that the proportion of attention directed to salient facial features (eye, nose and mouth) differs between emotions (Eisenbarth and Alpers, 2011). Finally, we expected that more attention is directed to salient features for emotions that are more difficult to rate i.e., have a lower accuracy rate.

## 2 Material and methods

### 2.1 Procedure

Individuals without a (self-reported) neurological or psychiatric disorder, aged 18–65, were recruited among staff of two healthcare institutions using flyers and on social media (i.e., Facebook groups and Twitter). Participants were informed by the researchers and signed informed consent. They received €10 compensation for participating. In a single ±90-minute session, participants completed a demographic questionnaire and three emotion recognition tasks; a photo, video and VR task. The order of administration was randomized. Participants were randomized to complete the VR task either in a VR environment with a low or a high number of environmental distractors. Ethical approval was given by the ethics committee of the University of Groningen Psychology department.

### 2.2 Measures

#### 2.2.1 Photo task - Facial Emotional Expressions: Stimuli and Tests (FEEST) (Young et al., 2002)

The FEEST is a 10-minute computerized task consisting of 60 pictures portraying the six basic emotions (anger, disgust, fear, happiness, sadness or surprise, see Fig. 2). Faces are displayed for 5 s after which participants decide which emotion was shown.

#### 2.2.2 Video task – Bell-Lysaker Emotion Recognition Test (BLERT; Dutch version) (Bryson et al., 1997)

The BLERT consists of 35 ten-second video fragments in which actors speak emotionally ambiguous sentences (Fig. 2). Through body language, facial expression and intonation, one of the basic emotions, or a neutral expression, is expressed. Participants have to indicate which emotion was portrayed. The task takes about 8 min.

#### 2.2.3 VR emotion recognition task

**Fig. 1:**
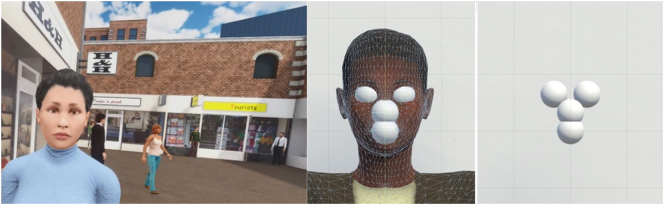
The VR street environment, and AOI placement on the avatars. AOIs were scaled for the avatar's face size.

The VR task took place in a VR street environment created by CleVR, where participants rated emotions of virtual characters (avatars) (Fig. 1) (Nijman et al., 2019). VR was presented through the Oculus Rift DK2 with an integrated eye-tracker (SensoMotoric Instruments) and headphones with ambient street noises. Participants navigated the street by altering their body orientation and operating a joystick enabling forward and backward movement.

(Source: CleVR.)

**Fig. 2:**
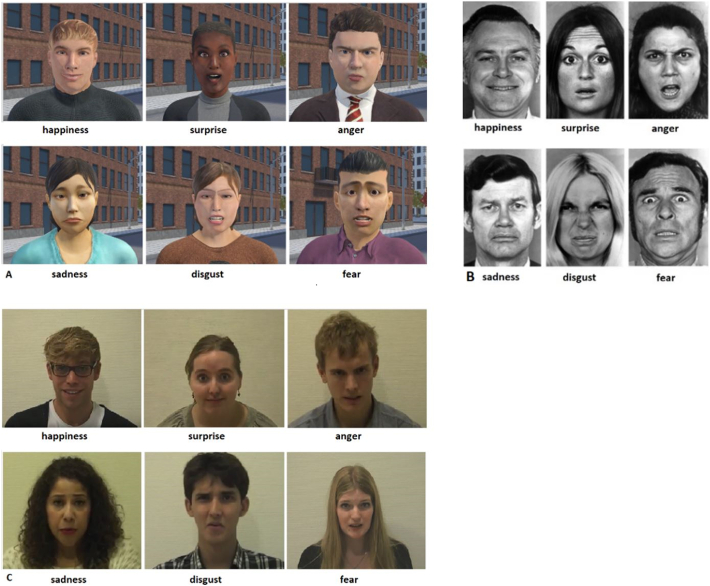
Examples of emotion stimuli of the A) VR task, B) photo task (FEEST) and C) video task (BLERT).

**Fig. 3:**
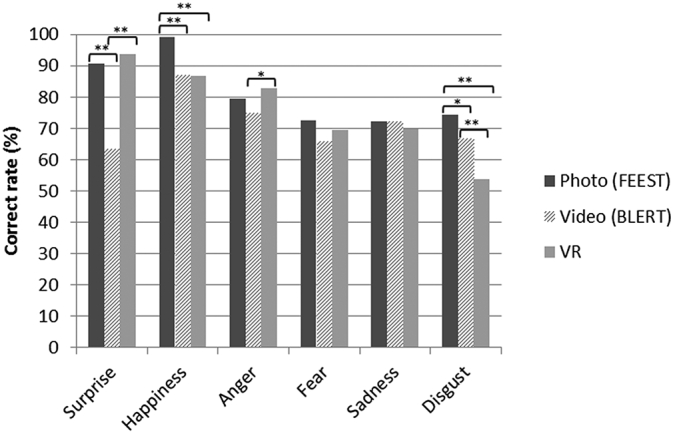
Recognition rate for each emotion recognition task. Pairwise comparison significant at *p < 0.05 or **p < 0.001.

**Fig. 4:**
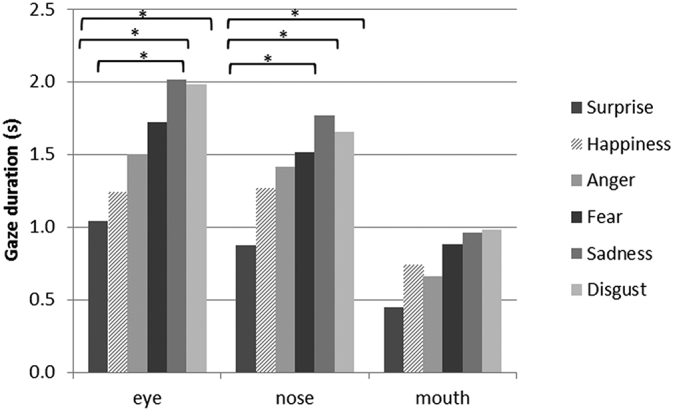
Absolute gaze duration for the total sample per emotion and AOI. Pairwise comparison significant at *p < 0.05.

Twenty avatars were standing at random locations in the VR street environment. When a participant moved within a two-meter radius, the avatar oriented its position towards the participant and displayed an emotion (anger, disgust, fear, happiness, sadness, surprise, or neutral) for 10 s. Simultaneously, a selection screen popped up, displaying four random answer options; one correct answer and three incorrect answer options. Answers could be selected with the joystick. The screen provided feedback by turning green (correct) or red (wrong). In case of a wrong answer, a second chance was given.

The number of avatars in the streets was manipulated; in the low environmental distraction condition no additional avatars were walking through the streets, whereas in the high environmental distraction condition 22 neutral-looking avatars were walking leisurely through the streets. The task takes approximately 12 min.

### 2.3 Eyetracking

During the VR task, eye movement of both eyes was recorded with a 75 Hz HMD integrated eye-tracker (SMI). Before the start of the VR task, a 9-point calibration was performed. Areas of interest (AOI) were predefined for the eyes, nose and mouth with the limited-radius Voronoi tessellation method, which was found to be the most objective and robust method for face stimuli (Fig. 1) (Hessels et al., 2016). AOIs were programmed to register when a participant viewed an AOI. No fixation points were used prior to the stimuli. Attention, operationalized as gaze duration was calculated per AOI and emotion. Relative gaze was also calculated in percentages per AOI and emotion. Relative gaze was calculated by dividing the gaze duration to a specific AOI by the total time the AOIs of an avatar (eyes, nose and mouth) were viewed per emotion. Eye-tracking data were checked for possible drift by comparing gaze duration during the first and second half of the experiment. In case of drift or insufficient calibration, the eye-tracking data were not used.

### 2.4 Statistics

Analyses were conducted using SPSS 24. Significance was accepted at p < 0.05. Emotion accuracy rates (percentage correct) were compared with RM-ANOVA or non-parametric Kruskal–Wallis tests. For the VR task, only responses to the first attempt were used in analyses. Associations between recognition accuracy, age, sex and education were explored with regression analysis for each task. A confusion matrix for correct and incorrect responses was made and percentages of occurrence were reported. The low and high VR distraction conditions were compared on accuracy with *t*-tests or non-parametric Mann-Whitney *U* tests.

Eye-tracking data were analyzed with RM-ANOVA on emotion (anger, disgust, fear, happiness, sadness and surprise), AOI (eyes, nose and mouth) and distraction condition (low and high). If the sphericity assumption was violated, the Greenhouse-Geisser correction was applied. For further analysis of differences between emotions, one-way ANOVAs were performed. Post-hoc pairwise comparisons were Bonferroni corrected.

## 3 Results

In total 100 participants were included (M_age_ = 37.1, SD_age_ = 12.3), of whom 57 were female. Participants had varying education levels: 23% vocational, 18% higher secondary, 33% higher tertiary and 26% higher tertiary/university degree. Data were missing at random due to nausea in VR (n = 1) and technical issues (FEEST n = 1; BLERT n = 4; VR task n = 4).

### 3.1 Emotion recognition

For the VR task, recognition accuracy was highest for surprise (93.7%). Happiness (86.7%) and anger (82.7%) also had high accuracy ratings. Sadness (69.7%), fear (69.6%) and disgust (53.8%) had the lowest recognition accuracy. No significant differences in accuracy were found between the high and low distraction conditions.

Emotion accuracy rates were rather similar for the three tasks (Fig. 3). RM-ANOVAs identified some differences in accuracy between the VR task and the photo (FEEST) and video (BLERT) tasks. Compared to the VR task, happiness and disgust of the FEEST, and disgust of the BLERT had higher recognition accuracy. Anger and surprise were rated significantly better in the VR than video task.

The confusion matrix shows that disgust was most commonly confused with anger in all tasks (Table 1). Whereas fear was confused mostly with surprise in VR and photographs. For the video task, fear was confused most commonly with sadness. A tendency to confuse sadness with neutral was found in both VR and video-rated faces. For photographs, neutral was not an option, and sadness was mainly confused with fear and disgust. Anger was mainly confused with disgust, surprise (except for the video task), or neutral.

**Table 1:** Confusion matrix showing correct and incorrect responses in percentage for each task.

		Correct answer					
		Happiness	Surprise	Anger	Sadness	Fear	Disgust
VR task							
Selected answer	Happiness	**86.7**	3.9	1.1	1.1	1.6	1.1
	Surprise	3.9	**93.7**	4.6	2.1	15.6	2.9
	Anger	0.7	1.1	**82.7**	3.5	0.8	28.5
	Sadness	1.8	0.0	0.0	**69.7**	6.1	5.0
	Fear	0.4	1.1	1.8	2.5	**69.6**	5.8
	Disgust	0.4	0.4	4.6	3.2	3.2	**53.8**
	Neutral	6.3	0.0	5.3	18.0	3.2	2.9

Photo task (FEEST)							
Selected answer	Happy	**99.3**	1.0	0.1	0.0	0.1	0.0
	Surprise	0.6	**90.8**	8.0	4.6	18.4	1.0
	Anger	0.0	0.2	**79.4**	2.6	0.7	22.8
	Sad	0.1	0.2	1.2	**72.3**	1.6	1.3
	Fear	0.0	7.0	2.5	10.7	**72.5**	0.5
	Disgust	0.0	0.7	8.8	9.8	6.6	**74.4**

Video task (BLERT)							
Selected answer	Happiness	**86.9**	14.8	0.2	0.2	1.7	0.4
	Surprise	5.6	**63.5**	1.0	0.8	6.7	3.5
	Anger	0.0	4.0	**74.8**	0.2	1.3	17.5
	Sadness	0.4	1.0	2.1	**72.3**	10.2	5.2
	Fear	0.2	1.7	0.6	12.5	**65.8**	1.9
	Disgust	0.0	6.3	15.0	0.6	6.5	**66.7**
	Neutral	6.9	8.8	6.3	13.3	6.3	4.8

Recognition accuracy was not predicted by sex or education in any of the three emotion recognition tasks. For the BLERT, age significantly predicted accuracy (b = −0.38, p < 0.001); further analysis revealed that this age-effect was present for sad, disgust and fear. Age also influenced the accuracy of the VR task (b = −0.59, p < 0.001); with every ten years of age, emotion recognition performance decreased on average by 5.9%. Analysis per emotion revealed that this age-effect was consistently present for all emotions, except for surprise.

### 3.2 Eye-tracking

Fifty participants had good quality eye-tracking data (M_age_ = 35.7, SD_age_ = 12.4; 48% female). Data were missing due to inadequate calibration (n = 25), wearing soft contact lenses/glasses (n = 13), technical problems (n = 6), incompletion of task (n = 3), cybersickness (n = 2) and drift (n = 1).

For absolute gaze duration (Fig. 4 and Table 2), the RM-ANOVA showed a main effect of AOI (F(2,96) = 12.4; p < 0.01). Post-hoc comparisons revealed that significantly more time was spent looking at the eyes (M = 1.58; SE = 0.14) and nose area (M = 1.42; SE = 0.11) than the mouth (M = 0.78; SE = 0.12). Also, a main effect of type of emotion was observed (F(5,184) = 17.7; p < 0.01). In general, surprised and happy faces were viewed the shortest, and disgust and sad faces the longest.

**Table 2:** Means and standard deviations of the gaze duration per emotion and distraction condition.

	Low distraction (n = 25)				High distraction (n = 25)				∆Total_high-low_
	Eyes	Nose	Mouth	Total	Eyes	Nose	Mouth	Total	
Absolute gaze duration in seconds									
Surprise	0.86 (0.56)	0.88 (0.54)	0.51 (0.47)	2.25 (0.83)	1.22 (0.79)	0.87 (0.47)	0.39 (0.39)	2.48 (1.08)	0.23
Happiness	1.11 (0.71)	1.29 (0.84)	0.91 (0.94)	3.30 (1.26)	1.37 (1.13)	1.24 (0.73)	0.56 (0.70)	3.18 (1.67)	−0.12
Anger	1.19 (0.88)	1.37 (0.85)	0.79 (0.81)	3.35 (1.49)	1.81 (1.64)	1.46 (0.71)	0.53 (0.52)	3.80 (2.28)	0.45
Fear	1.37 (0.77)	1.49 (1.36)	0.96 (1.21)	3.82 (2.22)	2.07 (1.17)	1.54 (0.96)	0.80 (0.93)	4.41 (2.12)	0.59
Disgust	1.45 (0.83)	1.62 (1.14)	1.12 (1.16)	4.19 (1.92)	2.51 (2.17)	1.69 (0.84)	0.84 (0.90)	5.05 (2.83)	0.86
Sadness	1.89 (1.70)	1.89 (1.70)	1.10 (1.72)	4.87 (3.28)	2.14 (1.64)	1.65 (0.99)	0.83 (0.91)	4.62 (2.70)	−0.25
Total	1.31 (1.02)	1.42 (1.16)	0.90 (1.12)		1.86 (1.53)	1.41 (0.84)	0.66 (0.76)		

Relative gaze duration in %									
Surprise	38.0 (23.3)	37.8 (16.3)	24.2 (24.4)		47.0 (20.2)	34.8 (10.6)	18.3 (17.3)		
Happiness	36.1 (21.7)	38.3 (15.7)	25.6 (21.4)		42.3 (23.2)	39.8 (16.6)	17.8 (17.7)		
Anger	35.9 (21.4)	40.2 (17.1)	23.9 (21.7)		42.6 (21.1)	41.8 (13.1)	15.6 (13.6)		
Fear	41.3 (22.5)	36.7 (15.7)	22.1 (21.0)		49.5 (19.3)	33.9 (12.6)	16.6 (17.1)		
Disgust	38.3 (20.4)	36.8 (14.4)	24.9 (19.2)		47.9 (18.7)	35.2 (11.9)	16.8 (14.0)		
Sadness	41.8 (25.8)	38.5 (16.0)	19.7 (20.7)		46.3 (21.0)	36.4 (14.7)	17.3 (15.7)		
Total	38.8 (22.3)	38.0 (15.6)	23.4 (21.1)		45.9 (20.5)	36.9 (13.5)	17.1 (15.7)		

There was a marginally significant interaction between emotion and AOI (F(6,282) = 2.1; p = 0.06). ANOVAs per AOI revealed that more time was spent at the eyes for disgust and sadness compared to surprise. Additionally, significantly more time was spent at the eyes for sadness than happiness. Further, participants spent less time looking at the nose area while viewing surprised faces compared to sad, fearful and disgusted faces. For the mouth, no differences between emotions were observed. A marginally significant interaction of AOI and distraction condition (F(2,81) = 2.8; p = 0.08) was found; during the high distraction condition, more time was spent looking at the eyes and less at the mouth as compared to the low distraction condition.

For relative gaze, a significant main effect of AOI (F(2,77) = 15.0; p < 0.001) was found. Post-hoc comparisons showed that gaze was directed for larger proportions of time towards the eyes (M = 42.2%; SE = 2.8%) and nose (M = 37.5%; SE = 1.7%) compared to the mouth (M = 20.2%; SE = 2.5%). There was a significant interaction between emotion and AOI (F(10,480) = 3.7; p = 0.00). Post-hoc comparisons showed that for anger and happiness, a smaller proportion of time attention was directed to the eyes and more to the nose compared to the other emotions.

## 4 Discussion

We investigated a novel VR emotion recognition task intended for neuropsychiatric assessment and training. Our findings support the validity of the VR task; emotion recognition accuracy and confusion patterns in virtual faces were very similar to those of real faces in photos and videos, except for disgust, which was recognized less accurately in VR. There was an age effect; younger people rated virtual faces more accurately. Eye-tracking revealed that attention was directed predominantly to the eyes and nose, and relatively less attention was directed to the eyes when looking at happy and angry faces compared to other emotions.

The similarity between virtual and real face tasks supports the validity and utility of the VR task and also supports the utility of *emotional* avatars in general for training and interventions, such as VR-based CBT (Nijman et al., 2019; Pot-Kolder et al., 2018; Klein Tuente et al., 2018). As the field of VR is expanding rapidly, validated emotional expressions will be of major relevance for future clinical applications.

### 4.1 Virtual versus real emotions

The direct comparison of three tasks within a large sample is an important strength of this study. When considering differences between tasks, findings were remarkably similar. The tasks differed in the number of stimuli, presentation method, presence of verbal information, color, intensity and dynamics. Though it may seem more convenient to use photos or videos if they yield the same information, VR offers important advantages. VR tasks can easily be personalized and enable interaction (e.g., gaze direction, verbal interaction) (Nijman et al., 2019). Further, incorporation of immersive environments can enhance ecological validity, and facilitate practice within environments resembling real-life situations.

Consistent with prior research, we found that recognition accuracy was highest for surprise and happiness, followed by sadness and anger, and lowest for fear and disgust (Calvo and Nummenmaa, 2016). Disgust was the only emotion that did not reach satisfying recognition in virtual faces compared to the photo and video task. However, similar to real faces, virtual disgust was predominantly confused with anger. This limitation of disgust recognition in virtual faces is well documented (Gutiérrez-Maldonado et al., 2013; Dyck et al., 2008; Fabri et al., 2002). Though marked progress has been made, with recognition accuracy improving from 20 to 55% in a decade (Gutiérrez-Maldonado et al., 2013; Calvo et al., 2018; Spencer-Smith et al., 2001), it has been suggested that further advancement may be made by improving wrinkling at the base of the nose (Dyck et al., 2008). Alternatively, it has been argued that disgust represents a mixture of emotions instead of a basic emotion (Kohler et al., 2004). This may cause difficulty in both creating and identifying disgust, and perhaps recognition rates around 60–70% are optimal for disgust, as shown in the conventional tasks.

Confusion patterns in virtual faces strongly resembled patterns in real faces. In accordance with the review of Calvo and Nummenmaa (2016), fear was most commonly mistaken for surprise, disgust for anger, and sadness for both disgust and neutral. Unexpectedly, VR sadness was predominantly confused with neutral and not disgust, which might be attributed to the aforementioned non-optimal presentation of disgust in VR.

In the current study, age was negatively associated with emotion recognition accuracy in the VR and video tasks. Comparable age-related declines have been found for another 2D VR emotion recognition task (Dyck et al., 2008). Computer game exposure was suggested as an explanation. However, gaming did not fully explain age-related declines, as even after correcting for gaming experience, an age-effect remained (Dyck et al., 2008). Further, we found that gender and education level were not associated with accuracy in any of the tasks. While subtle advantages of females and higher education levels have been reported in the general population, findings are inconsistent (Bediou et al., 2007; Kret and De Gelder, 2012; Meletti et al., 2009; Kessels et al., 2014). Positive associations between education and emotion perception have been found for depression and bipolar disorder, but not for schizophrenia (Kohler et al., 2011; Kohler et al., 2010). This suggests that better neurocognitive skills or intelligence might play a compensatory role that is disorder-specific.

### 4.2 Environmental distractors and eye-tracking

No differences in accuracy were found when more environmental distractors were present, even though a higher number of stimuli may be more demanding of cognitive capacity. Possibly, healthy people have ample cognitive capacity to process the number of stimuli, or the impact of distractors may have been small due to the neutrality of the stimuli (Wieser and Brosch, 2012). Eye-tracking data did show some potentially interesting patterns. For 4/6 emotions, gaze was directed longer at salient facial features in crowded streets (marginally significant). More specifically, gaze was directed on average 0.5 s longer to the eyes in crowded streets. This could mean that in more challenging situations, the eye region is of more importance.

In line with previous research, positive emotions required the least attention to salient facial features, respectively 2.4 s for surprise and 3.2 s for happiness (Calvo and Nummenmaa, 2016; Wells et al., 2016). Low recognition accuracies of disgust, sadness and fear were reflected in longer gaze durations (±4.5 s) (Wells et al., 2016; Eisenbarth and Alpers, 2011; Calvo et al., 2018). This illustrates that during unconstrained exploration, people take a substantial amount of time before decision-making and suggests that for training purposes, even larger or unrestrained time windows may be appropriate.

Attention was directed longer to the eyes and nose than the mouth. This difference in the eye-mouth region is consistent with findings in real faces (Wells et al., 2016; Eisenbarth and Alpers, 2011). Although the amount of attention drawn to the nose area may seem large, this area contains part of the nasolabial area and cheeks which contains information through wrinkling and widening of the nostrils. Similarly, Calvo et al. (2018) reported that approximately 40% of the time gaze was directed to the nose area while rating emotions in videos.

For sadness and disgust, attention was directed to the eyes and nose longer compared to the well-recognized surprise; also, happiness and sadness differed significantly. These findings emphasize the importance of the eyes. Attention generally lingers on the eyes longer than on other areas, such as the mouth, and as emotions become more difficult to recognize, this relative difference becomes more pronounced (Eisenbarth and Alpers, 2011). Interestingly, a similar trend was found for environmental distractors; with more avatars present in the environment, on average 8.5% more attention was drawn to the eyes.

Concerning relative gaze duration, participants directed gaze to the eyes for a shorter percentage of time for angry and happy faces than other emotions. Consistently with prior research, the eyes appear of reduced importance for these expressions. When happy faces were viewed, a slightly (non-significantly) higher percentage (range: 0–4%) attention was directed to the mouth compared to other emotions. Initially, we expected more information to be conveyed by the mouth for happiness due to smiling. An explanation for this could be that recognition of smiling might be so easy that it barely takes any time. Alternatively, virtual mouths may lack certain laugh wrinkles. However, similar results to our study were found with a photo task, and the authors argued that the mouth and eyes are equally valuable for happy recognition (Wells et al., 2016).

### 4.3 Limitations

General limitations are the use of self-report for eye problems (e.g., eye acuity, and stereoblindness), neurological and psychiatric disorders. The photo, video and VR tasks differ substantially in how emotions are presented, e.g., color versus black-and-white, different intensity of emotions and sound. This limits conclusions on what aspects of the tasks contributed to the differences in emotion recognition scores. However, we chose these tasks as we wanted to compare the VR task with existing, validated tasks that are commonly used in clinical practice. Further, the option neutral was not present in the FEEST. Nevertheless, as the FEEST shows high-intensity emotions we expect the influence of neutral to be marginal (Calvo and Nummenmaa, 2016; Wells et al., 2016).

A major limitation of the current research was caused by constraints of the current software. In the VR task, participants were offered only four emotions to choose from, which could influence accuracy scores as by random guess people had a 25% chance in the VR task to answer correctly, in contrast to the photo task, where this chance was 16.6%. Further, feedback was provided and two chances were given. Even though only the first attempts were used for analyses, this could have caused learning effects. To check if participants' emotion recognition accuracy improved over time (i.e., investigate if there were any learning effects during the trial) we analyzed differences in emotion recognition accuracy between the first and second half (each half thus consisting of 10 items) of the VR task. The average accuracy rate was 74% during the first half and 76% during the second half of the task, showing that learning effects within the trial were minimal. Furthermore, we checked the percentage correct for second attempts. During second attempts surprise and happiness were also recognized best: surprise 94.4% (n = 18), happiness 89.5% (n = 38), anger 82.4% (n = 51), disgust 80.7% (n = 176), fear 79.5% (n = 117), and sadness 78.2% (n = 87).

Finally, VR eye-tracking needs further development; at the time the study was conducted, it was impossible to continuously register eye-tracking in addition to the VR environment, as this was graphically too demanding. Therefore, AOIs were pre-programmed, reducing possibilities for detailed analyses of visual attention towards environmental distractors. Furthermore, the eye-tracking system could register only at a frequency of 75 Hz, which is a relatively low frequency and prevents accurate detection of other measures such as saccades. Though caution needs to be taken with saccades as cybersickness was found to influence saccases (Cebeci et al., 2019).

### 4.4 Future research

Future research will need to investigate the VR emotion recognition task in patient samples in which emotion recognition problems are common, such as patients with psychosis, autism and neurological damage. The environment is expected to have a larger impact on emotion recognition skills in patients with a neurological or psychiatric condition, due to impairments in cognition and attention. Such information is of relevance for how we train emotion recognition skills, as current interventions often use isolated faces as practice stimuli, even though in real life recognition takes place in highly complex and demanding situations. Additionally, research on the cognitive and neural mechanisms underlying the processing of virtual and real emotional faces is needed. Using eye-tracking in the different kinds of tasks (photo, video and VR) can be a first step. Finally, implicit physiological measurements such as pupil dilation may provide valuable insights into the processing of emotion stimuli (Cebeci et al., 2019; Snowden et al., 2016; Chen et al., 2017).

## 5 Conclusion

Immersive VR seems a promising method for facial emotion recognition. Recognition patterns were similar in virtual and real faces. This is of clinical importance for current and future interventions, and research using “emotional” avatars, as it indicates that emotions of virtual faces can be used as stimuli. Currently, such VR stimuli are used, among other things, for social cognition training, and cognitive behavior therapy for anxiety and psychosis (Nijman et al., 2019; Pot-Kolder et al., 2018; Freeman et al., 2019). Furthermore, neuropsychiatric assessment and training can benefit from the possibilities of VR to expose people to dynamic emotions within social contexts relevant to daily life.

## Funding

This research did not receive any specific grant from funding agencies in the public, commercial, or not-for-profit sectors.

## Declaration of competing interest

The authors declare that they have no known competing financial interests or personal relationships that could have appeared to influence the work reported in this paper.
